# The association between CYP1A1 genetic polymorphisms and coronary artery disease in the Uygur and Han of China

**DOI:** 10.1186/1476-511X-13-145

**Published:** 2014-09-05

**Authors:** Jin-Guo Zou, Yi-Tong Ma, Xiang Xie, Yi-Ning Yang, Shuo Pan, Dilare Adi, Fen Liu, Bang-Dang Chen

**Affiliations:** Department of Cardiology, First Affiliated Hospital of Xinjiang Medical University, Urumqi, 830054 People’s Republic of China; Xinjiang Key Laboratory of Cardiovascular Disease Research, Urumqi, 830054 People’s Republic of China

**Keywords:** *CYP1A1*, Single nucleotide polymorphism, Coronary artery disease, Case–control study

## Abstract

**Background:**

The cytochrome P450, family 1, subfamily A, polypeptide 1 (*CYP1A1*) gene is expressed in the vascular endothelium, which metabolizes arachidonic acid into 20-hydroxyeicosatetraenoic acid (20-HETE) and epoxyeicosatrienoic acids (EETs). 20-HETE mediates cardiovascular homeostasis and growth response in vascular smooth muscle cells (VSMCs) as well as the anti-platelet effect. EETs are potent endogenous vasodilators and inhibitors of vascular inflammation. This study assessed the association between human *CYP1A1* gene polymorphisms and coronary artery disease (CAD) in the Uygur and Han in China.

**Methods:**

Two independent case–control studies that recruited Han (389 patients with CAD and 411 controls) and Uygur participants (293 patients with CAD and 408 controls) analyzed the relationship between *CYP1A1* single nucleotide polymorphisms (SNPs: rs4886605, rs12441817, rs4646422 and rs1048943) and CAD. All patients with CAD and controls were genotyped for the four SNPs of *CYP1A1* using TaqMan SNP genotyping assays.

**Results:**

In the Uygur group, the distribution of the dominant model(CC vs CT + TT) of rs4886605 for the total sample and the males was significantly different between CAD patients and control participants (P = 0.001 and P = 0.012, respectively), The difference remained significant after a multivariate adjustment (P = 0.018, P = 0.015, respectively). The rs12441817 was also associated with CAD in a dominant model for all participants (P = 0.003) and men (P = 0.012), and the difference remained significant after a multivariate adjustment (P = 0.016, P = 0.002, respectively). However, we did not observe differences in the Uygur females and Han group with regard to the allele frequency or genotypic distribution of rs4886605 and rs12441817 between patients with CAD and control participants. Patients with CAD did not significantly differ from the control participants with regard to the distributions of rs4646422 and rs1048943 genotypes, the dominant model, the recessive model, or allele frequency in the Han and Uygur groups.

**Conclusion:**

Both rs4886605 and rs12441817 SNPs of the CYP1A1 gene are associated with CAD in the Uygur population of China.

## Introduction

Coronary artery disease (CAD) accounts for nearly 40% of all the Causes of mortality in developed countries [1, 2]. CAD is a complex, multifactorial and polygenic disorder thought to result from the interaction between an individual’s genetic makeup and various environmental factors [3]. Various gene variants are associated with CAD [4, 5].

Cytochrome P450 (CYP) is a super-family of cysteino-heme enzymes that mediate the oxidative metabolism of exogenous and endogenous molecules [6]. CYP enzymes play an important role in maintaining cardiovascular health because they catalyze the formation and/or metabolism of several endogenous molecules, such as cholesterol, androgens, estrogens, and several arachidonic acid metabolites, that affect several cardiovascular functions [7, 8]. Mounting evidence demonstrates that CYP enzymes are involved in the pathogenesis of CAD [9, 10]. Polymorphisms of CYP genes such as, CYP2C8, CYP2C9, CYP2J2 (epoxyeicosatrienoic acid[EET] synthesis) [11–13], CYP8A (prostacyclin synthesis) [14], CYP11B2 (aldosterone synthesis) [15], CYP17, and CYP19 (sex hormone synthesis) are related to CAD [16]. The human gene cytochrome P450, family 1, subfamily A, polypeptide 1 (CYP1A1) is expressed in the vascular endothelium. In addition to the roles that CYP1A1 plays in metabolizing exogenous compounds such as polycyclic aromatic hydrocarbons (PAHs) and aromatic amines that increase the development of atherosclerotic lesions, it can metabolize arachidonic acid to terminal 20-hydroxyeicosatetraenoic acid (20-HETEs; 75–90%) and, to a lesser extent, epoxyeicosatrienoic acids (EETs; 5–7%) [17–19]. 20-HETE plays critical roles in the regulation of cardiovascular, renal and pulmonary homeostasis as well as the growth response in vascular smooth muscle cells (VSMCs), cardiac function and vascular tone [7]. In addition, 20-HETE has beneficial anti-platelet effects and inhibits sodium reabsorption in the renal tubules [20]. EETs generally have cardioprotective effects [1].

Recently, several reports investigated the association between CYP1A1 genetic polymorphisms and the risk of CAD associated with cigarette smoking. Wang et al. [9], Manfredi et al. [21], and Cornelis et al. [10], studied the association between the CYP1A1 MspI polymorphism, cigarette smoking, and the risk of CAD. They provided inconsistent results. According to the physiological and pathological mechanism of CYP1A1, the interaction between CYP1A1 and smoking can cause atherosclerosis. furthermore, this gene can metabolize arachidonic acid into 20-HETE and EETs, which can directly lead to atherosclerosis [1]; However, few studies have excluded the factors related to smoking and independently assessed the relationship between CYP1A1 and coronary heart disease.

The present case–control study aimed to assess the association between the human gene CYP1A1 and CAD excluding the risk factors related to smoking in the Uygur and Han population who are two major ethnic groups in Xinjiang, China. It can be further clarified physiological and pathological mechanism that CYP1A1 can cause CAD through Arachidonic acid metabolites such as 20-HETE and EETs.

## Materials and methods

### Ethical approval of the study protocol

Written informed consent was obtained from all participants, who explicitly provided permission for all DNA analyses and the collection of relevant clinical data. The Ethics Committee of the First Affiliated Hospital of Xinjiang Medical University (Urumqi, China) approved this study, which was conducted according to the standards of the Declaration of Helsinki.

### Participants

The participants were recruited from the Han and Uygur population who live in the Xinjiang Uygur Autonomous Region of China. All patients and controls received differential diagnoses for chest pain at the Cardiac Catheterization Laboratory of the First Affiliated Hospital of Xinjiang Medical University between 2006 and 2013. Highly skilled physicians performed all coronary angiography procedures using the Judkins approach [22]. At least two experienced imaging specialists interpreted the coronary angiography findings, and the final CAD diagnosis was made based on the angiography report.

We randomly sampled 293 Uygur patients with CAD and 408 ethnically and geographically matched participants for the control group. In addition, we randomly sampled 389 Han patients with CAD and 411 ethnically and geographically matched participants for the control group. CAD was defined as the presence of at least one significant coronary artery stenosis of more than 50% luminal diameter according to coronary angiography for all groups. All control participants also underwent a coronary angiogram and did not have coronary artery stenoses or show clinical or electrocardiogram evidence of myocardial infarction (MI) or CAD. Control participants were not healthy individuals; some were diagnosed with hypertension, DM or hyperlipidemia. Thus, the control group was exposed to the same risk factors of CAD; however, their coronary angiogram results were normal. Data and information regarding traditional coronary risk factors including hypertension, diabetes mellitus (DM), and smoking were collected from all study participants. The diagnosis of hypertension was established if patients were on antihypertensive medication or if the mean of 3 measurements of systolic blood pressure (SBP) was >140 mm Hg or diastolic blood pressure (DBP) >90 mm Hg, respectively. Diabetes mellitus was diagnosed using the criteria of the World Health Organization (WHO). In addition, individuals with a fasting plasma glucose of >7.0 mmol/L, or with a history of diabetes or treatment with insulin were considered as diabetic. The smoking variable was dichotomized as smokers (including current and ex-smokers) and non-smokers. All patients with impaired renal function, malignancy, connective tissue disease, valvular disease or chronic inflammatory disease were excluded.

### Blood collection and DNA extraction

Fasting blood samples drawn via venipuncture in the catheter room were taken from all participants before cardiac catheterization. The blood samples were drawn into a 5-mL ethylene diamine tetraacetic acid (EDTA) tube and centrifuged at 4000 × g for 5 min to separate the plasma content. Genomic DNA was extracted from the peripheral leukocytes using the standard phenol-chloroform method. The DNA samples were stored at -80°C until use. For use, the DNA was diluted to a concentration of 50 ng/μL.

### Genotyping

**Figure 1:**
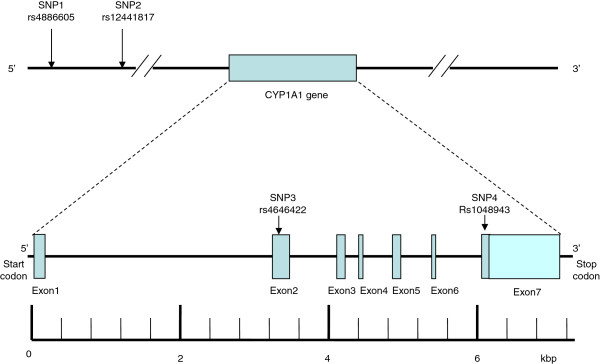
**Structure of the human** ***CYP1A1*** **gene.** The gene consists of seven exons (boxes) separated by six introns (lines; intergenic regions). Filled boxes indicate the coding regions, while arrows indicate the locations of single nucleotide polymorphism (SNPs). kbp, kilobase pairs.

CYP1A1 is located on chromosome 15q24. This gene consists of approximately 6.068 kilobase pairs (kbp) and contains seven exons that are separated by six introns (Figure 1). A total of 287 SNPs are listed for the human CYP1A1 gene in the National Center for Biotechnology Information SNP database (http://www.ncbi.nlm.nih.gov/SNP). Using Haploview 4.2 and the HapMap phase II database, we obtained four tag SNPs (rs4886605, rs12441817,rs4646422 and rs1048943) using minor allele frequency (MAF) of >0.1 and linkage disequilibrium patterns with r^2^ ≥ 0.5 as a cut off. The position of SNP1, SNP2, SNP3 and SNP4 (rs4886605, rs12441817, rs4646422 and rs1048943) occurred in order of decreasing distance from the CYP1A1 gene 3′end (Figure 1). SNP1 (rs4886605) was located 8.037 kbp upstream from the start codon in exon 1; SNP2 (rs12441817) was located 7.863 kbp upstream from the start codon in exon 1; SNP3 (rs4646422) was observed in the exon 2 region of the gene; and SNP4 (rs1048943) was observed in the exon 7 region of the gene. Genotyping was undertaken using the TaqMan^®^ SNP Genotyping Assay (Applied Biosystems). The primers and probes used for the TaqMan^®^ SNP Genotyping Assays (ABI) were chosen based on information on ABI’s website (http://appliedbiosystems.com.cn/). Thermal cycling was conducted using the Applied Biosystems 7900HT Standard Real-Time PCR System. Plates were read using the Sequence Detection Systems (SDS) automation controller software v2.4 (ABI). PCR amplification was performed using 2.5 μL of TaqMan Universa Master Mix, 0.15 μL probes and 1.85 ddH2O in a 6-μL final reaction volume containing 1 μL DNA. Thermal cycling conditions were as follows: 95°C for 5 min; 40 cycles of 95°C for 15 s; and 60°C for 1 min. All 96-well plates were read using Sequence Detection Systems (SDS) automation controller software v2.4 (ABI).

### Biochemical analyses

Serum concentrations of total cholesterol (TC), triglyceride (TG), glucose (Glu), high-density lipoprotein cholesterol (HDL-C), and low-density lipoprotein cholesterol (LDL-C), were measured using standard methods at the Central Laboratory of the First Affiliated Hospital of Xin jiang Medical University.

### Statistical analyses

All continuous variables (e.g., age, BMI, pulse, and cholesterol levels) are presented as the means ± standard deviation (S.D.). The differences between the CAD and control groups were analyzed using independent-samples *T*-test. The differences with regard to the frequencies of smoking, hypertension, diabetes mellitus, and CYP1A1 genotypes were analyzed using *χ*^2^ test or Fisher’s exact test where appropriate. The Hardy-Weinberg equilibrium was assessed using a *χ*^2^ analysis. Logistic regression analyses with effect ratios (odds ratios [ORs] and 95% CIs) were used to assess the contribution of the major risk factors. All statistical analyses were performed using SPSS 16.0 for Windows (SPSS Institute, Chicago, USA). P-value is 2-tailed, and P-values of less than 0.05 were considered significant.

## Results

Table 1 shows the clinical characteristics of the Han and Uygur study participants. for total,men and women, The plasma concentrations of Glu and LDL-C as well as the rates of EH, DM, and smoking were all significantly higher for patients with CAD than control participants in the two ethnic population, and the plasma concentrations of Glu was significantly higher only for total and men group. BMI was significantly higher among total, men and women patients with CAD than their control counterparts in Uygur populations, and the plasma concentration of TC was significantly higher for women group.

**Table 1:** **Han and Uygur population characteristics of study participants**

	Han									Uygur								
	Total			Men			Women			Total			Men			Women		
	CAD	Control	P value	CAD	Control	P value	CAD	Control	P value	CAD	Control	P value	CAD	Control	P value	CAD	Control	P value
Number (n)	389	411		294	224		95	187		293	408		210	205		83	203	
Age (years)	58.73 ± 7.32	57.82 ± 8.53	0.502	59.82 ± 8.24	58.52 ± 8.43	0.332	58.21 ± 6.37	59.76 ± 6.76	0.052	56.97 ± 5.58	55.93 ± 5.59	0.086	56.57 ± 8.33	54.96 ± 9.06	0.09	54.20 ± 3.89	52.30 ± 4.83	0.076
BMI (kg/m2)	27.62 ± 3.71	28.23 ± 2.74	0.428	26.63 ± 1.72	26.34 ± 1.75	0.201	30.63 ± 1.78	30.17 ± 2.23	0.069	32.34 ± 7.20	28.11 ± 4.37	<0.001^*^	31.45 ± 6.84	28.48 ± 3.98	<0.001^*^	32.97 ± 7.40	26.73 ± 5.41	<0.001^*^
Pulse(beats/min)	73.82 ± 10.23	73.13 ± 10.01	0.673	74.24 ± 10.52	73.76 ± 10.23	0.324	73.13 ± 9.24	72.45 ± 8.53	0.802	74.59 ± 10.92	76.56 ± 7.93	0.38	73.49 ± 11.20	74.76 ± 8.45	0.732	76.91 ± 9.74	80.38 ± 5.17	0.339
Glu (mmol/L)	6.12 ± 2.34	5.28 ± 1.67	<0.001^*^	5.63 ± 1.68	5.42 ± 1.45	0.036^*^	6.48 ± 3.12	5.42 ± 1.59	<0.001^*^	6.60 ± 2.78	5.24 ± 2.38	<0.001^*^	6.21 ± 2.64	5.20 ± 2.55	<0.001^*^	7.31 ± 3.25	5.37 ± 2.41	<0.001^*^
TG (mmol/L)	1.97 ± 1.22	1.82 ± 1.76	0.978	1.74 ± 0.87	1.58 ± 0.69	0.284	2.23 ± 1.64	1.62 ± 1.18	0.254	1.83 ± 1.25	1.76 ± 1.35	0.435	1.77 ± 0.99	1.83 ± 1.50	0.638	2.10 ± 2.07	1.70 ± 1.24	0.061
TC (mmol/L)	4.35 ± 1.08	4.12 ± 0.74	0.262	4.35 ± 1.17	4.26 ± 0.82	0.431	4.62 ± 1.23	4.52 ± 0.85	0.873	4.24 ± 1.08	4.15 ± 0.98	0.34	4.18 ± 1.06	4.13 ± 0.97	0.565	4.50 ± 1.15	4.17 ± 0.97	0.037^*^
HDL (mmol/L)	1.14 ± 0.23	1.26 ± 0.31	0.054	1.12 ± 0.26	1.19 ± 0.47	0.672	1.15 ± 0.36	1.33 ± 0.24	0.528	0.98 ± 0.13	1.01 ± 0.12	0.142	0.97 ± 0.31	0.98 ± 0.25	0.738	1.044 ± 0.20	1.04 ± 0.10	0.702
LDL (mmol/L)	2.53 ± 0.79	2.16 ± 0.85	<0.001^*^	2.62 ± 0.84	2.56 ± 0.68	0.005^*^	2.58 ± 1.02	2.24 ± 0.58	<0.001^*^	2.86 ± 0.86	2.59 ± 0.97	<0.001^*^	2.90 ± 0.84	2.56 ± 0.99	<0.001^*^	2.84 ± 0.87	2.72 ± 0.89	0.356
EH (%)	59.32	47.63	0.023^*^	62.31	43.34	<0.001^*^	57.27	51.22	0.031^*^	42.68	18.42	<0.001^*^	34.35	20.01	<0.001^*^	51	16.53	<0.001^*^
DM (%)	27.23	13.56	<0.001^*^	22.74	15.03	<0.001^*^	31.18	12.26	<0.001^*^	26.71	12.84	<0.001^*^	21.53	7.73	<0.001^*^	30.69	17.95	<0.001^*^
Smoke (%)	58.48	31.43	<0.001^*^	87.59	56.38	<0.001^*^	12.13	3.62	0.002^*^	33.15	17.97	<0.001^*^	63.73	34.9	<0.001^*^	5.63	1.23	0.023^*^

BMI, body mass index; Glu, glucose; TG, triglyceride; TC, total cholesterol; HDL, high density lipoprotein; LDL, low density lipoprotein; EH, essential hypertension; DM, diabetes mellitus. Continuous variables were expressed as the means ± standard deviations. The P-values of continuous variables were calculated using independent-samples T-tests. The P-values of categorical variables were calculated using X ^2^ test. *P < 0.05.

Tables 2 and 3 show the distribution of the genotypes and alleles for the four CYP1A1 gene SNPs in the Han and Uygur populations. The genotypic distribution of each SNP matched the predicted Hardy-Weinberg equilibrium values for both ethnicities (P > 0.05 for the CAD and control groups; data not shown).

**Table 2:** **Genotype and allele distributions in the control subjects and patients with CAD in the Han population**

	Total			Men			Women		
Variants	CAD n (%)	Control n (%)	P Value	CAD n (%)	Control n (%)	P Value	CAD n (%)	Control n (%)	P Value
rs4886605(SNP1)									
Genotyping									
CC	79(20.3%)	96(23.4%)		58(19.7%)	51(22.8%)		21(22.1%)	45(24.1%)	
CT	190(48.8%)	200(48.7%)		146(49.7%)	119(53.1%)		44(46.3%)	81(43.3%)	
TT	120(30.8%)	115(28.0%)	0.494	90(30.6%)	54(24.1%)	0.248	30(31.6%)	61(32.6%)	0.881
Dominant model									
CC	79(20.3%)	96(23.4%)		58(19.7%)	51(22.8%)		21(22.1%)	45(24.1%)	
TT + CT	310(79.7%)	315(76.6%)	0.297	236(80.3%)	173(77.2%)	0.4	74(77.9%)	142(75.9%)	0.713
Recessive model									
TT	120(30.8%)	115(28.0%)	0.494	90(30.6%)	54(24.1%)	0.248	30(31.6%)	61(32.6%)	
CC + CT	269(69.2%)	296(72.0%)	0.373	204(69.4%)	170(75.9%)	0.102	65(68.4%)	126(67.4%)	0.86
Additive model									
CT	190(48.8%)	200(48.7%)		146(49.7%)	119(53.1%)		44(46.3%)	81(43.3%)	
CC + TT	199(51.1%)	211(51.3%)	0.959	148(50.3%)	105(46.9%)	0.434	51(53.7%)	106(56.7%)	0.632
Allele									
C	348(44.7%)	392(47.7%)		262(44.6%)	221(49.3%)		86(45.3%)	171(45.7%)	
T	430(55.3%)	430(52.3%)	0.236	326(55.4%)	227(50.7%)	0.127	104(54.7%)	203(54.3%)	0.918
rs12441817(SNP2)									
Genotyping									
CC	83(21.3%)	83(20.2%)		65(22.1%)	35(15.6%)		18(18.9%)	48(25.7%)	
CT	205(52.7%)	199(48.4%)		154(52.4%)	121(54.0%)		51(53.7%)	78(41.7%)	
TT	101(26.0%)	129(31.4%)	0.235	75(25.5%)	68(30.4%)	0.141	26(27.4%)	61(32.6%)	0.153
Dominant model									
TT	101(26.0%)	129(31.4%)	0.235	75(25.5%)	68(30.4%)	0.141	26(27.4%)	61(32.6%)	
CC + CT	288(74.0%)	282(68.6%)	0.09	219(74.5%)	156(69.6%)	0.222	69(72.6%)	126(67.4%)	0.367
Recessive model									
CC	83(21.3%)	83(20.2%)		65(22.1%)	35(15.6%)		18(18.9%)	48(25.7%)	
TT + CT	306(78.7%)	328(79.8%)	0.691	229(77.9%)	189(84.4%)	0.064	77(81.1%)	139(74.3%)	0.208
Additive model									
CT	205(52.7%)	199(48.4%)		154(52.4%)	121(54.0%)		51(53.7%)	78(41.7%)	
CC + TT	184(47.3%)	212(51.6%)	0.226	140(47.6%)	103(46.0%)	0.712	44(46.3%)	109(58.3%)	0.056
Allele									
C	371(47.7%)	365(44.4%)		284(48.3%)	191(42.6%)		87(45.8%)	174(46.5%)	
T	407(52.3%)	457(55.6%)	0.188	304(51.7%)	257(57.4%)	0.07	103(54.2%)	200(53.5%)	0.869
rs4646422(SNP3)									
Genotyping									
AA	270(69.4%)	286(69.6%)		206(70.1%)	152(67.9%)		64(67.4%)	134(71.7%)	
AG	102(26.2%)	115(28.0%)		74(25.2%)	67(29.9%)		28(29.5%)	48(25.7%)	
GG	17(4.4%)	10(2.4%)	0.294	14(4.8%)	5(2.2%)	0.187	3(3.2%)	5(2.7%)	0.759
Dominant model									
AA	270(69.4%)	286(69.6%)		206(70.1%)	152(67.9%)		64(67.4%)	134(71.7%)	
GG + AG	119(30.6%)	125(30.4%)	0.957	88(30.0%)	72(32.1%)	0.59	31(32.6%)	53(28.4%)	0.457
Recessive model									
GG	17(4.4%)	10(2.4%)	0.294	14(4.8%)	5(2.2%)	0.187	3(3.2%)	5(2.7%)	
AA + AG	372(95.6%)	401(97.6%)	0.129	280(95.2%)	219(97.8%)	0.129	92(96.8%)	182(97.3%)	0.818
Additive model									
AG	102(26.2%)	115(28.0%)		74(25.2%)	67(29.9%)		28(29.5%)	48(25.7%)	
AA + GG	287(73.8%)	296(72.0%)	0.576	220(74.8%)	157(70.1%)	0.23	67(70.5%)	139(74.3%)	0.496
Allele									
A	642(82.5%)	687(83.6%)		486(82.7%)	371(82.8%)		156(82.1%)	316(84.5%)	
G	136(17.5%)	135(16.4%)	0.573	102(17.3%)	77(17.2%)	0.946	34(17.9%)	58(15.5%)	0.468
rs1048943(SNP4)									
Genotyping									
CC	20(5.1%)	17(4.1%)		15(5.1%)	8(3.6%)		5(5.3%)	9(4.8%)	
CT	152(39.1%)	148(36.0%)		115(39.1%)	80(35.7%)		37(38.9%)	68(36.4%)	
TT	217(55.8%)	246(59.9%)	0.47	164(55.8%)	136(60.7%)	0.45	53(55.8%)	110(58.8%)	0.888
Dominant model									
TT	217(55.8%)	246(59.9%)		164(55.8%)	136(60.7%)		53(55.8%)	110(58.8%)	
CC + CT	172(44.2%)	165(40.1%)	0.244	130(44.2%)	88(39.3%)	0.26	42(44.2%)	77(41.2%)	0.626
Recessive model									
CC	20(5.1%)	17(4.1%)		15(5.1%)	8(3.6%)		5(5.3%)	9(4.8%)	
TT + CT	369(94.9%)	394(95.9%)	0.499	279(94.9%)	216(96.4%)	0.402	90(94.7%)	178(95.2%)	0.87
Additive model									
CT	152(39.1%)	148(36.0%)		115(39.1%)	80(35.7%)		37(38.9%)	68(36.4%)	
CC + TT	237(60.9%)	263(64.0%)	0.371	179(60.9%)	144(64.3%)	0.429	58(61.1%)	119(63.6%)	0.671
Allele									
C	192(24.7%)	182(22.1%)		145(24.7%)	96(21.4%)		47(24.7%)	86(23.0%)	
T	586(75.3%)	640(77.9%)	0.231	443(75.3%)	352(78.6%)	0.223	143(75.3%)	288(77.0%)	0.645

CAD, coronary artery disease; SNP, single-nucleotide polymorphism.

**Table 3:** **Genotype and allele distributions in control subjects and patients with CAD in the Uygur population**

	Total			Men			Women		
Variants	CAD n (%)	Control n (%)	P Value	CAD n (%)	Control n (%)	P Value	CAD n (%)	Control n(%)	P Value
rs4886605(SNP1)									
Genotyping									
CC	75(25.60%)	153(37.50%)		52(24.80%)	74(36.10%)		23(27.70%)	79(38.90%)	
CT	162(55.30%)	191(46.80%)		121(57.60%)	104(50.70%)		41(49.40%)	87(42.90%)	
TT	56(19.10%)	64(15.70%)	0.004^*^	37(17.60%)	27(13.20%)	0.036^*^	19(22.90%)	37(18.20%)	0.193
Dominant model									
CC	75(25.60%)	153(37.50%)		52(24.80%)	74(36.10%)		23(27.70%)	79(38.90%)	
TT + CT	218(74.40%)	255(62.50%)	0.001^*^	158(75.20%)	131(63.90%)	0.012^*^	60(72.30%)	124(61.10%)	0.073
Recessive model									
TT	56(19.10%)	64(15.70%)		37(17.60%)	27(13.20%)		19(22.90%)	37(18.20%)	
CC + CT	237(80.90%)	344(84.30%)	0.235	173(82.40%)	178(86.80%)	0.21	64(77.10%)	166(81.80%)	0.367
Additive model									
CT	162(55.30%)	191(46.80%)		121(57.60%)	104(50.70%)		41(49.40%)	87(42.90%)	
CC + TT	131(44.70%)	217(53.20%)	0.027^*^	89(42.40%)	101(49.30%)	0.159	42(50.60%)	116(57.10%)	0.313
Allele									
C	312(53.24%)	497(60.91%)		225(53.57%)	252(61.46%)		87(52.41%)	245(60.34%)	
T	274(46.76%)	319(39.09%)	0.006^*^	195(46.43%)	158(38.54%)	0.021^*^	79(47.59%)	161(39.66%)	0.081
rs12441817(SNP2)									
Genotyping									
CC	43(14.70%)	45(11.00%)		30(14.30%)	20(9.80%)		13(15.70%)	25(12.30%)	
CT	150(51.20%)	178(43.60%)		110(52.40%)	92(44.90%)		40(48.20%)	86(42.40%)	
TT	100(34.10%)	185(45.30%)	0.01^*^	70(33.30%)	93(45.40%)	0.034^*^	30(36.10%)	92(45.30%)	0.348
Dominant model									
TT	100(34.10%)	185(45.30%)		70(33.30%)	93(45.40%)		30(36.10%)	92(45.30%)	
CC + CT	193(65.90%)	223(54.60%)	0.003^*^	140(66.70%)	112(54.60%)	0.012^*^	53(63.90%)	111(54.70%)	0.154
Recessive model									
CC	43(14.70%)	45(11.00%)		30(14.30%)	20(9.80%)		13(15.70%)	25(12.30%)	
TT + CT	250(85.30%)	363(89.00%)	0.151	180(85.70%)	185(90.20%)	0.156	70(84.30%)	178(87.70%)	0.449
Additive model									
CT	150(51.20%)	178(43.60%)		110(52.40%)	92(44.90%)		40(48.20%)	86(42.40%)	
CC + TT	143(48.80%)	230(56.40%)	0.048^*^	100(47.60%)	113(55.10%)	0.126	43(51.80%)	117(57.60%)	0.368
Allele									
C	236(40.27%)	268(32.84%)		170(40.48%)	132(32.20%)		66(39.78%)	136(33.50%)	
T	350(59.73%)	548(67.16%)	0.004^*^	250(59.52%)	278(67.8%)	0.013^*^	100(60.22%	270(66.5%)	0.155
rs4646422(SNP3)									
Genotyping									
AA	251(85.7%)	350(85.80%)		178(84.80%)	177(86.30%)		73(88.00%)	173(85.20%)	
AG	39(13.30%)	53(13.00%)		32(15.20%)	26(12.70%)		7(8.40%)	27(13.30%)	
GG	3(1.00%)	5(1.20%)	0.963	0(0.00%)	2(1.00%)	0.319	3(3.60%)	3(1.50%)	0.291
Dominant model									
AA	251(85.70%)	350(85.80%)		178(84.80%)	177(86.30%)		73(88.00%)	173(85.20%)	
GG + AG	42(14.30%)	58(14.20%)	0.965	32(15.20%)	28(13.70%)	0.647	10(12.00%)	30(14.80%)	0.546
Recessive model									
GG	3(1.00%)	5(1.20%)		0(0.00%)	2(1.00%)		3(3.60%)	3(1.50%)	
AA + AG	290(99.00%)	403(98.80%)	0.803	210(100.00%)	203(99.00%)	0.243	80(96.40%)	200(98.50%)	0.275
Additive model									
AG	39(13.30%)	53(13.00%)		32(15.20%)	26(12.70%)		7(8.40%)	27(13.30%)	
AA + GG	254(86.70%)	355(87.00%)	0.901	178(84.80%)	179(87.30%)	0.453	76(91.60%)	176(86.70%)	0.248
Allele									
A	541(92.32%)	753(92.28%)		388(92.38%)	380(92.68%)		153(92.17%)	373(91.87%)	
G	45(7.68%)	63(7.72%)	0.977	32(7.62%)	30(7.31%)	0.869	13(7.83%)	33(8.13%)	0.906
rs1048943(SNP4)									
Genotyping									
CC	5(1.70%)	11(2.70%)		3(1.40%)	5(2.40%)		2(2.40%)	6(3.00%)	
CT	85(29.00%)	112(27.50%)		63(30.00%)	54(26.30%)		22(26.50%)	58(28.60%)	
TT	203(69.30%)	285(69.90%)	0.642	144(68.60%)	146(71.20%)	0.562	59(71.10%)	139(68.50%)	0.899
Dominant model									
TT	203(69.30%)	285(69.90%)		144(68.60%)	146(71.20%)		59(71.10%)	139(68.50%)	
CC + CT	90(30.70%)	123(30.20%)	0.872	66(31.40%)	59(28.70%)	0.557	24(28.90%)	64(31.60%)	0.664
Recessive model									
CC	5(1.70%)	11(2.70%)		3(1.40%)	5(2.40%)		2(2.40%)	6(3.00%)	
TT + CT	288(98.30%)	397(97.40%)	0.387	207(98.60%)	200(97.50%)	0.452	81(97.60%)	197(97.10%)	0.797
Additive model									
CT	85(29.00%)	112(27.50%)		63(30.00%)	54(26.30%)		22(26.50%)	58(28.60%)	
CC + TT	208(71.00%)	296(72.60%)	0.651	147(70.00%)	151(73.60%)	0.408	61(73.50%)	145(71.50%)	0.724
Allele									
C	95(16.21%)	134(16.42%)		69(16.43%)	64(15.61%)		26(15.66%)	70(17.24%)	
T	491(83.79%)	682(83.58%)	0.916	351(83.57%)	346(84.39%)	0.748	140(84.33%)	336(82.76%)	0.647

CAD, coronary artery disease; SNP, single-nucleotide polymorphism; *P < 0.05.

The distribution of the SNP1 (rs4886605) genotypes (P = 0.004) and the additive model (P = 0.027) significantly differed between the CAD and control participants in the entire Uygur sample. The distribution of SNP1 (rs4886605) alleles and the dominant model (CC vs CT + TT) significantly differed between CAD and control participants (alleles: P = 0.006 [total sample] and P = 0.021 [men]; dominant model: P = 0.001 [total sample] and P = 0.012 [men]). The T allele of rs4886605 was found significantly more frequently in patients with CAD than controls (total: 46.76% vs 39.09%; men: 46.43% vs 38.54%). The dominant model (CC vs CT + TT) of rs4886605 was significantly lower among patients CAD than controls (total: 25.60% vs 37.50%; men: 24.80% vs 36.10%). The distribution of SNP2 (rs12441817) genotypes (P = 0.010) and the additive model (P = 0.048) significantly differed between patients with CAD and controls for the entire sample. The distribution of SNP2 (rs12441817) alleles and the dominant model (TT vs CC + CT) significantly differed between patients CAD and controls (alleles: P = 0.004 [total sample] and P = 0.013 [men]; dominant model: P = 0.003 [total sample] and P = 0.012 [men]). The C allele of rs12441817 was found significantly more frequently in patients with CAD than control participants (total: 40.27% vs 32.84%; men: 40.48% vs 32.20%). The dominant model (TT vs CC + CT) of rs12441817 was significantly lower among patients with CAD than control participants (total: 34.10% vs 45.30%; men: 33.30% vs 45.40%). Moreover, significant differences were not observed between patients with CAD and control participants (for total participants, males or females) in the Uygur group with regard to the distributions of rs4646422 or rs1048943, the dominant model, the recessive model, or allele frequency (P > 0.05). Similarly, significant differences were not observed between patients with CAD and control participants (for total participants, males or females) within the Han group with regard to the distributions of rs4886605, rs12441817, rs4646422 or rs1048943, the dominant model, the recessive model, or allele frequency (P > 0.05).

Tables 4 and 5 show that multiple logistic regression analyses were performed with TG, TC, HDL, Glu, LDL-C, EH, DM, and smoking because these variables are the major confounding factors for CAD. Table 4: The significant difference observed with regard to rs4886605 was retained after adjustment for TG, TC, HDL, Glu, LDL-C, EH, DM, and smoking within the Uygur population (total participants: OR = 0.368, 95% confidence intervals [CI] = 0.185–0.530, P = 0.018; men: OR = 0.350, 95% CI: 0.235–0.568, P = 0.015). Table 5: The significant difference observed with regard to rs12441817 was retained after multivariate adjustment for TG, TC, HDL, Glu, LDL-C, EH, DM, and smoking within Uygur population (total participants: OR = 0.253, 95% confidence intervals [CI] = 0.231–0.546, P = 0.016; men: OR = 0.241, 95% CI = 0.132–0.478, P = 0.002).

**Table 4:** **Multiple logistic regression analysis for CAD patients and control subjects of Uygur population (rs4886605)**

	Total			Men			Women		
	OR	95% CI	P	OR	95% CI	P	OR	95% CI	P
Dominant model (CC vs CT + TT)	0.368	0.185–0.530	0.018	0.35	0.235–0.568	0.015	0.626	0.321–1.412	0.204
Smoking	8.29	5.365–12.846	<0.001	10.254	5.713–18.375	<0.001	0.232	0.039–1.825	0.262
EH	6.651	3.957–10.952	<0.001	9.81	4.94–20.513	<0.001	4.103	1.875–9.184	<0.001
DM	1.524	0.949–2.495	0.284	1.902	0.926–3.854	0.087	0.95	0.358–2.243	0.757
Glu	1.352	1.241–1.482	<0.001	1.348	1.183–1.517	<0.001	1.485	1.253–1.762	<0.001
TG	1.347	1.273-1.638	0.251	1.384	1.169-1.615	0.015	1.653	1.321-1.859	0.021
TC	1.86	1.765-1.913	0.031	1.846	1.713-1.917	0.013	1.89	1.694-2.132	0.027
HDL	0.25	0.086-0.541	0.032	0.231	0.069-0.182	0.021	0.516	0.285-0.930	0.012
LDL	0.985	0.897–1.153	0.068	0.924	0.897–1.358	0.21	0.427	0.203–0.947	0.135

CAD, coronary artery disease; EH, essential hypertension; DM, diabetes mellitus; Glu, glucose; TG, triglyceride; TC, total cholesterol; HDL, high-density lipoprotein; LDL, Low-density lipoprotein.

**Table 5:** **Multiple logistic regression analysis for CAD patients and control subjects of Uygur population(rs12441817)**

	Total			Men			Women		
	OR	95% CI	P	OR	95% CI	P	OR	95% CI	P
Dominant model (TT vs CT + CC)	0.253	0.231–0.546	0.016	0.241	0.132–0.478	0.002	0.723	0.273–1.523	0.362
Smoking	9.152	6.278–13.373	<0.001	11.286	6.325–19.178	<0.001	0.471	0.357–1.932	0.378
EH	7.435	3.835–10.637	<0.001	9.513	4.631–20.278	<0.001	5.158	1.532–9.259	<0.001
DM	1.338	0.754–2.269	0.376	1.723	0.785–3.832	0.173	0.632	0.209–2.371	0.952
Glu	1.432	1.341–1.573	<0.001	1.409	1.283–1.672	<0.001	1.538	1.327–1.849	<0.001
TG	1.475	1.348-1.721	0.032	1.469	1.258-1.732	0.016	1.727	1.486-2.327	0.011
TC	1.532	1.467-1.853	0.012	1.563	1.427-1.725	0.028	1.542	1.329-1.935	0.045
HDL	0.305	0.132-0.529	0.041	0.327	0.235-0.473	0.035	0.273	0.075-0.683	0.037
LDL	0.653	0.532–1.243	0.072	0.736	0.658–1.235	0.237	0.639	0.347–0.953	0.375

CAD, coronary artery disease; EH, essential hypertension; DM, diabetes mellitus; Glu, glucose; TG, triglyceride; TC, total cholesterol; HDL, high-density lipoprotein; LDL, Low-density lipoprotein.

## Discussion

We found that variation in CYP1A1 gene is associated with CAD in the Uygur population of China. After multivariate adjustment, the associations between CYP1A1 gene polymorphisms with CAD were not modified. Our study is the first case–control study to investigate the association between the human CYP1A1 gene and CAD in the Uygur and Han populations in western China.

Several CYP enzyme families have been identified in the heart, endothelium, and smooth muscle of blood vessels. A link between the expression and activity of CYP and cardiovascular diseases (CVDs) such as hypertension, CAD, heart failure, stroke, cardiomyopathy, and arrhythmia has been established [7]. CYP1A1 polymorphisms can affect the metabolism of arachidonic acid, thereby resulting in an altered generating capacity of 20-HETE and EETs . 20-HETE have 5 physiological functions. First, 20-HETE increases the intracellular calcium concentration and regulates the vasoconstrictor responses of angiotensin II, vasopressin, and norepinephrine [23–25]. Second, 20-HETE increases thymidine incorporation in cells and regulates growth responses in vascular smooth muscle cells (VSMCs) in response to norepinephrine and angiotensin II [26–28]. Third, 20-HETE inhibits Na + -K + -ATPase activity and sodium transport in the proximal tubule and is a key mediator of the long-term control of arterial pressure [29–31]. Fourth, 20-HETE significantly contributes to ischemia -reperfusion injury, and the exogenous administration of 20-HETE significantly increases infarct size [32, 33]. Fifth, 20-HETE inhibits platelet aggregation and the formation of thromboxane A2 (TxA2) during platelet activation [34]. in addition, CYP1A1 also metabolize arachidonic acid to EETs, which are potent endogenous vasodilators, inhibitors of vascular inflammation, and possessors of potent vasodilatory and antiapoptotic and anti-thrombotic properties in the cardiovascular system [35].

Several genetic polymorphisms have been reported in the CYP1A1 gene including T6235C and T5639C in the 3′-flanking region, A4889G and C4887A at exon 7 [36]. Functional studies have revealed that these polymorphisms are associated with increased CYP1A1 activity and/or inducibility [37]. Wang et al. [9] reported significant association between CYP1A1 MspI polymorphism and the risk of coronary artery disease(CAD) in cigarette smoking. This increased risk was more evident among light smokers with OR of 3.44, but not observed in non-smokers or heavy smokers. Nevertheless, Manfredi et al. [21] found no significant association was observed between CYP1A1 MspI polymorphism and the presence of CAD, or the number of significantly diseased vessels in smokers. In agreement with this study, Cornelis et al. [10] reported no significant association between CYP1A1 polymorphism and the risk of myocardial infarction (MI) in a study from Costa Rica. Chih-Ching Yeh et al. [38] report CYP1A1*2C polymorphism, particularly in non-smokers, may be associated with the individual susceptibility to CAD. From the above example, we have considered Association between CYP1A1 and CAD was different on account of polymorphisms of the CYP1A1 gene, Ethnic differences, environmental factors. CYP1A1 might cause CAD in some smokers or non-smokers. Their mechanisms of CYP1A1 were different. CYP1A1 metabolites arachidonic acid to 20-HETE, EETs, which cause CAD, in non-smoking.

In this study, we hypothesized that variability in the CYP1A1 gene might affect the risk of CAD through CYP1A1 as an arachidonic acid epoxygenase enzyme in nonsmoking. We genotyped four SNPs of this gene in the Uygur and Han of China and assessed the association between CYP1A1 polymorphisms and CAD using a case–control analysis.

A significant difference was observed in the genotypic distribution of SNP1 (rs4886605) between the patients with CAD and controls within the full Uygur sample. When men and women were analyzed separately, the T allele frequency of rs4886605 was higher among men with CAD than their control counterparts. No such differences were observed among women. Thus, the risk of CAD is increased with regard to the T allele of rs4886605 in men. The dominant model (CC vs CT + TT) was significantly lower among patients with CAD than controls for all participants and men; furthermore, this difference was retained after adjusting for Glu, LDL-C, EH, DM, and smoking (Table 4) using multiple logistic regression analyses. This finding indicated that the risk of CAD was decreased with the presence of the CC genotype of rs4886605 in Uygur men. In the full Uygur sample, a significant difference was observed in the genotypic distribution of SNP2 (rs12441817) between patients with CAD and controls. When men and women were analyzed separately, the C allele of rs12441817 was more frequency in men with CAD than their control counterparts. No such differences were observed among women. Thus, the risk of CAD was increased with the appearance of the C allele in rs12441817 among men. The dominant model (TT vs CC + CT) was significantly lower among patients with CAD than controls for all participants and men; furthermore, this difference remained significant after adjusting for Glu, LDL-C, EH, DM, and smoking (Table 5). This finding indicates that the risk of CAD was decreased with the presence of the TT genotype in rs12441817 among Uygur men.

We found associations between CAD and the CYP1A1 SNPs rs4886605 and rs12441817 among Uygur men. This result might be caused by two reasons. First, it might be because of sex hormones; for instance, estrogen protects against oxidative stress and is vasoprotective [39–41]. The vascular protective effects of estradiol are metabolized into 2-hydroxyestradiol (2HE) by CYP1A1, and 2HE is converted into 2-methoxyestradiol (2ME) by catechol-O-methyl transferase. 2ME is antimitogenic, anti-angiogenic and pro-apoptotic [42]. Second, it might be because of less female samples. However, patients with CAD and controls participants in the Han group did not significantly differ with regard to the distributions of the rs4886605 and rs12441817 genotypes, the dominant model, the recessive model, or allele frequency (P > 0.05). This genetic difference associated with coronary heart disease might differ across ethnic groups because of race,diet, or lifestyle.

Although SNP4 (rs1048943) was observed in the exon 7 region of the CYP1A1 gene and the polymorphisms caused a loss of transcription factor binding at site Sp7, the synthesis of 20-HETE and EETs was reduced. Chih-Ching Yeh [38] showed that rs1048943 was independently associated with a decreased risk of coronary artery disease in non-smoker (OR = 0.32, 95% CIs = 0.15–0.70) in the Taiwanese population, But they didn’t find the association between rs1048943 and CAD in smoker. This study further indicated some polymorphisms of CYP1A1 can cause CAD through metabolism of 20-HETE and EETs excluding the risk factors like smoking in different ethnic groups. Our study was inconsistent with that study, showing no significant association between rs1048943 and CAD among non-smoking participants in the Uygur and Han of china. Differences in populations such as race, geographical and environment factors, might explain these results between Chinese and Taiwanese population.

Few studies have focused on the association between SNP3 (rs4646422) in the exon 2 and CAD . We did not find significant differences between the patients with CAD and controls with regard to the distributions of the rs4646422 genotypes, the dominant model, the recessive model, or allele frequency among the Han and Uygur samples.

Our study has several limitations. On one hand, the present study analyzed only a small sample data, More studies will need to incorporate a large samplesize for confirming the association. On the other hand, further studies will need to be undertaken in order to clarify the underlying molecular mechanism that polymorphism of CYP1A1 gene was associated with CAD .

## Conclusions

In conclusion, we found that rs4886605 and rs12441817 might be two novel polymorphisms of the CYP1A1 gene associated with CAD in the Uygur population in China. The CC genotype of rs4886605 and the TT genotype of rs12441817 in the CYP1A1 gene might be protective genetic markers of CAD, whereas the T allele of rs4886605 and the C allele of rs12441817 might be genetic risk markers of CAD in the Uygur population in China.
