# Erratum to: The association between CYP1A1 genetic polymorphisms and coronary artery disease in the Uygur and Han of China

**DOI:** 10.1186/s12944-015-0121-3

**Published:** 2015-09-28

**Authors:** Jin-Guo Zou, Yi-Tong Ma, Xiang Xie, Yi-Ning Yang, Shuo Pan, Dilare Adi, Fen Liu, Bang-Dang Chen

**Affiliations:** Department of Cardiology, First Affiliated Hospital of Xinjiang Medical University, Urumqi, 830054 People’s Republic of China; Xinjiang Key Laboratory of Cardiovascular Disease Research, Urumqi, 830054 People’s Republic of China

Although the focus of our article in Lipids in Health and Disease [1] reports novel data and has a different focus compared to publications in Clinical Biochemistry, Lipids in Health and Disease and Clinical and Applied Thrombosis/Hemostasis [2–5], we acknowledge that we have duplicated some text. We apologize for the inappropriate overlap between our two publications and our lack of transparency about the similarities between these articles.
